# Structural, Pro-Inflammatory and Calcium Handling Remodeling Underlies Spontaneous Onset of Paroxysmal Atrial Fibrillation in JDP2-Overexpressing Mice

**DOI:** 10.3390/ijms21239095

**Published:** 2020-11-30

**Authors:** Mariana S. Parahuleva, Jens Kockskämper, Jacqueline Heger, Wolfram Grimm, Anna Scherer, Sarah Bühler, Julian Kreutz, Rainer Schulz, Gerhild Euler

**Affiliations:** 1 Internal Medicine/Cardiology and Angiology, University Hospital of Giessen and Marburg, 35033 Marburg, Germany; grimmw@med.uni-marburg.de (W.G.); julian.kreutz@gmx.de (J.K.); 2 Biochemical-Pharmacological Centre (BPC) Marburg, Institute of Pharmacology and Clinical Pharmacy, University of Marburg, 35043 Marburg, Germany; jens.kockskaemper@staff.uni-marburg.de (J.K.); scherer6@students.uni-marburg.de (A.S.); buehlers@students.uni-marburg.de (S.B.); 3 Institute of Physiology, Justus Liebig University, 35392 Giessen, Germany; Jacqueline.Heger@physiologie.med.uni-giessen.de (J.H.); rainer.schulz@physiologie.med.uni-giessen.de (R.S.); gerhild.euler@physiologie.med.uni-giessen.de (G.E.)

**Keywords:** JDP2, atrial fibrillation, atrial remodeling, inflammation, calcium-handling proteins

## Abstract

Background: Cardiac-specific JDP2 overexpression provokes ventricular dysfunction and atrial dilatation in mice. We performed in vivo studies on JDP2-overexpressing mice to investigate the impact of JDP2 on the predisposition to spontaneous atrial fibrillation (AF). Methods: JDP2-overexpression was started by withdrawal of a doxycycline diet in 4-week-old mice. The spontaneous onset of AF was documented by ECG within 4 to 5 weeks of JDP2 overexpression. Gene expression was analyzed by real-time RT-PCR and Western blots. Results: In atrial tissue of JDP2 mice, besides the 3.6-fold increase of JDP2 mRNA, no changes could be detected within one week of JDP2 overexpression. Atrial dilatation and hypertrophy, combined with elongated cardiomyocytes and fibrosis, became evident after 5 weeks of JDP2 overexpression. Electrocardiogram (ECG) recordings revealed prolonged PQ-intervals and broadened P-waves and QRS-complexes, as well as AV-blocks and paroxysmal AF. Furthermore, reductions were found in the atrial mRNA and protein level of the calcium-handling proteins NCX, Cav1.2 and RyR2, as well as of connexin40 mRNA. mRNA of the hypertrophic marker gene ANP, pro-inflammatory MCP1, as well as markers of immune cell infiltration (CD68, CD20) were increased in JDP2 mice. Conclusion: JDP2 is an important regulator of atrial calcium and immune homeostasis and is involved in the development of atrial conduction defects and arrhythmogenic substrates preceding paroxysmal AF.

## 1. Introduction

Atrial fibrillation (AF) progresses from rare paroxysmal episodes, often unrecognized by patients, to long-lasting persistent and finally permanent stages [1]. Simultaneously, the atrium undergoes a complex remodeling process comprising electrical and functional/ contractile alterations of atrial cardiomyocytes, as well as structural changes of the extracellular matrix and vasculature, leading to a vicious circle that culminates in the perpetuation of the disease [2]. Key remodeling processes on the atrial myocyte level include a shortening of the action potential duration (APD) and altered intracellular calcium handling [3]. Furthermore, the structural atrial remodeling and later the arrhythmogenic substrate preceding spontaneous and persistent AF is atrial fibrosis [4]. Moreover, systemic or local/atrial inflammation has been proposed as a pivotal mediator that promotes AF-related fibrosis-mediated cellular changes [5].

Key proteins and mechanisms involved in these remodeling processes may include altered expression and/or regulation of sarcolemmal ion channels (e.g., potassium channels or the L-type Ca^2+^ channel, Cav1.2) causing reduction of APD; of calcium handling proteins (e.g., Na^+^/Ca^2+^ exchanger, NCX, or ryanodine receptor, RyR2) impairing intracellular calcium removal and release; of connexins (e.g., connexin40, connexin43) impairing intraatrial conduction; and of growth factors, extracellular matrix-regulating proteins and collagens underlying increased fibrosis [2]. Spontaneous sarcoplasmic reticulum (SR) calcium release via altered RyR2 may serve as a trigger for AF, with reduced APD, impaired conduction and increased fibrosis serving as substrates that promote the reentry and maintenance of AF.

The mechanisms underlying this extensive atrial remodeling are not fully understood. Moreover, the current treatment of AF is limited to rate control or prevention of stroke/transient ischemic attack with anticoagulation, or rhythm/electrical control during ablation and disruption of fibrosis-mediated cellular re-entry architecture, which misses to prevent the eventual perpetuation of the disease [1]. Therefore, the combination of basic research and clinical studies is needed to understand the pathophysiology of this intriguing arrhythmia, as well as to discover novel pharmaceutical targets and to improve the existing antiarrhythmic/ablation techniques.

Animal models that demonstrate characteristics of conduction defects and AF may have great potential to elucidate mechanisms that contribute to the development of AF [6]. Transgenic mice (TM) are an especially attractive mammalian animal model [6]. However, in many TM models, AF does not develop spontaneously under roaming conditions as it does in humans, but has to be induced by non-physiological conditions such as electrical burst pacing or anesthesia, which enhance the susceptibility to AF [6]. Therefore, TM models developing spontaneous AF in freely roaming mice are urgently needed.

JDP2-overexpressing mice might have the potential to serve as such a model. In these mice, the transcriptional repressor c-Jun dimerization protein 2 (JDP2) is overexpressed under the control of the cardiac specific α-MHC promoter [7]. Cardiac-specific overexpression of JDP2 increased atrial weight, atrial dilatation, atrial cardiomyocytes size and conduction defects in 4-week-old mice [7,8]. In a few of these mice AF was observed, although under anesthesia [8]. Moreover, JDP2 provoked ventricular contractile dysfunction within one week of overexpression [9] and JDP2 was identified as a novel, truly effective target for protection against cardiac remodeling and preserved cardiac function [10]. Very recently in our own studies on JDP2 mice, we were able to confirm atrial dilatation after 5 weeks of JDP2 overexpression [9]. These data gave us reason to analyze the development of conduction defects and AF in closer detail in JDP2 mice, and to characterize JDP2 mice as an animal model that spontaneously develops AF. To this end, we analyzed in this study the cardiac phenotype of JDP2 mice in 1- and 5-week-old mice. Electrocardiograms (ECGs) from awake mice indicate that JDP2 mice suffered from AF, the occurrence of which increased with increasing duration of JDP2 overexpression (from 1 to 5 weeks). Taken together, these data suggest a role of JPD2 in the development of a substrate for spontaneous onset of AF. Further insights on atrial electrical, structural and functional/contractile remodeling as an “atrial cardiomyopathy” in JDP2 mice were investigated to identify a potential role of this molecular platform for the development of AF. Thus, JDP2 mice may serve as an animal model in future research on initiating mechanisms of AF and testing therapeutic interventions (pharmaceutical and invasive/ablation procedures). Such research may reveal novel, truly effective targets for AF therapy and prevention.

## 2. Results

### 2.1. Conduction Defects and Arrhythmias in JDP2 Mice

**Figure 1:**
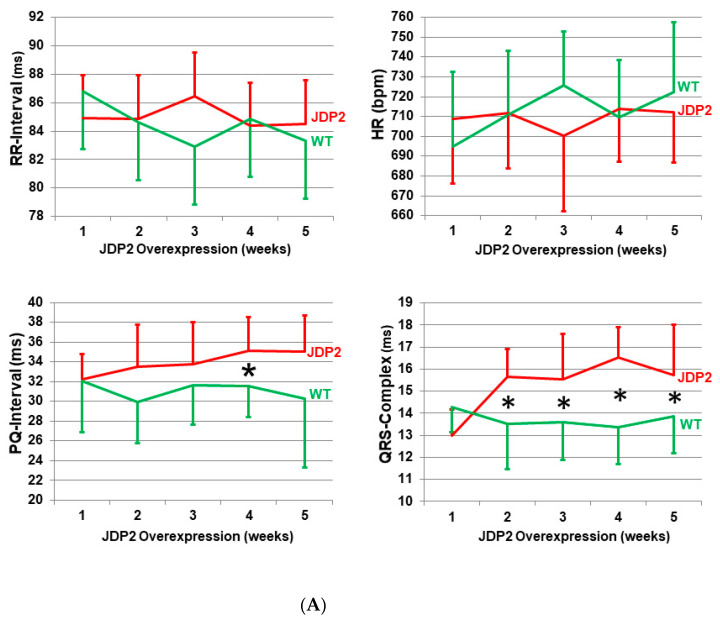

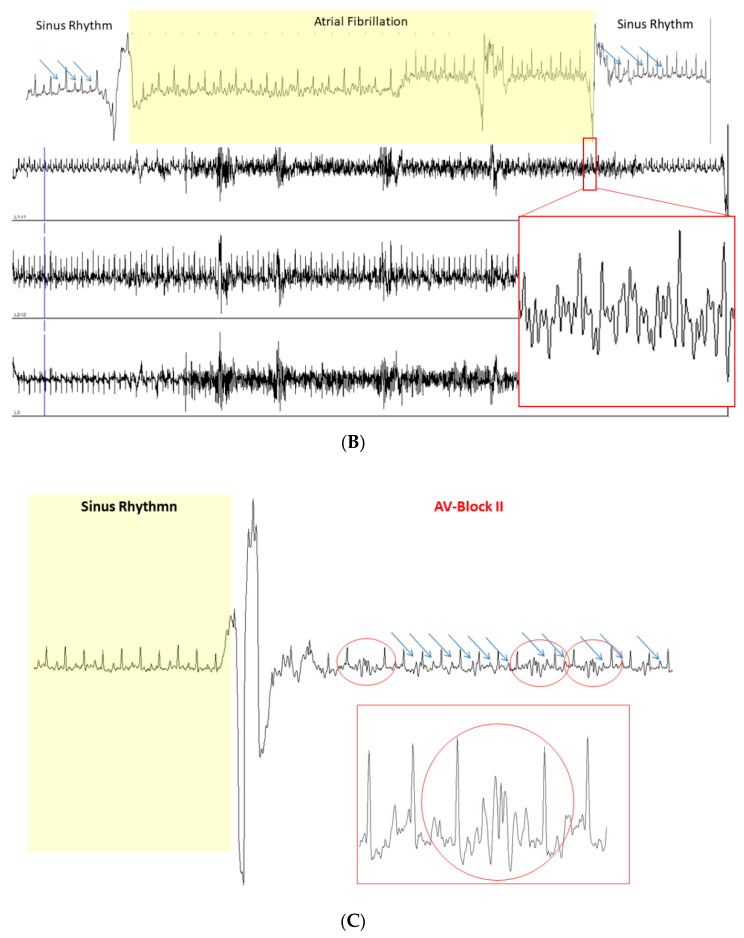
Conduction defects and atrial fibrillation (AF) detected in electrocardiogram (ECG) recordings. ECGs were recorded over 30 min in non-anesthetized mice after 1, 2, 3, 4 and 5 weeks of JDP2 overexpression. (**A**) After 2 weeks the PQ-interval was prolonged and QRS-complexes broadened. (*n* = 6–11, * *p* < 0.05 vs. WT). (**B**) Paroxysmal AF in 5-week-old JDP2 mice. ECG segments with sinus rhythm are presented (left and right white panel), and P-waves are indicated by small arrows. By contrast, other episodes (middle yellow panel) represent AF. The loss of P-waves and the irregularity of RR-intervals define an episode of AF. In derivation L1, the section in the red rectangle was magnified in order to clearly show the presence of rapid electrical activity. (**C**) Detection of second-degree AV Block Mobitz type II episodes in different magnifications (indicated in the red circles) after 4 and 5 weeks of JDP2 overexpression. P-waves are indicated by small arrows. Lead 1 from Einthoven is presented.

Up to the age of 4 weeks, JDP2 overexpression was repressed in JDP2 mice by feeding them a doxcycycline-containing diet. JDP2 overexpression started once doxycycline was omitted. Over 5 weeks, ECGs were recorded weekly. After one week of JDP2 overexpression, wild-type (WT) and JDP2 mice showed similar RR-intervals (84.9 ± 4.0 ms in JDP2 mice vs. 86.8 ± 5.0 ms in WT, *n =* 6–7, (not significant (n.s.)), P-wave durations (10.6 ± 0.8 ms in JDP2 mice vs. 10.0 ± 0.9 ms in WT., *n =* 6–7, n.s.), PQ-intervals (32.3 ± 2.6 ms in JDP2 mice vs. 32.0 ± 5.1 ms in WT, *n* = 6–7, n.s.) and QRS complexes (13.0 ± 1.2 ms in JDP2 mice vs. 14.3 ± 1.2 ms in WT, *n =* 6–7, n.s.) (Figure 1A, Table 1. Although mean values of RR-intervals and thereby heart rate remained unchanged over the entire observation period, calculation of heart rate variability revealed a non-significant trend of increased heart rate variabilities by about 30% in JDP2 mice (*n* = 6–11, n.s. vs. WT, Table 1). Furthermore, prolongation of PQ-intervals and QRS-complexes were observed after 2 weeks and prolonged JDP2 overexpression, reaching levels of 35.1 ± 3.6 ms in JDP2 mice vs. 30.3 ± 7.0 ms in WT for PQ-intervals, 11.6 ± 1.1 ms in JDP2 mice vs. 10.0 ± 1.5 ms in WT for P-wave duration (*n =* 10–11, *p* < 0.05), and 15.8 ± 2.3 ms in JDP2 mice vs. 13.9 ± 1.7 ms in WT for QRS-complexes (*n =* 10–11, *p* < 0.05) after 5 weeks of JDP2 overexpression (Figure 1A, Table 1). The increase in PQ-interval is an indicator of prolonged atrioventricular conduction time, which significantly increased after 4 weeks of JDP2 overexpression, indicating the development of first-degree atrioventricular block (AVB). In addition, second-degree AVB Mobitz type II was observed in JDP2 mice, as shown in Figure 1C. Similar to humans, AVBs in mice are considered predictors for the development of atrial fibrillation (AF) [11]. Prolonged P-wave duration is another predictor of AF in humans [12]. Indeed, spontaneous paroxysmal AF was detected in 9 of 11 JDP2 mice 4 and 5 weeks after the start of JDP2 overexpression (Figure 1B), whereas no episodes of AF were detected in WT mice (*n =* 10; *p* = 0.0002 vs. JDP2 mice, Fisher’s exact test). Furthermore, the maximum duration of paroxysmal AF in JDP2 mice 4 and 5 weeks after the start of JDP2 overexpression was less than 2 min. Moreover, the observed prolonged QRS-complex duration is possibly a more sensitive marker of increased left ventricle mass in JDP2 mice, which we have described previously [9]. In spite of the occurrence of AF after 4 and 5 weeks of JDP2 overexpression, mice did not display any increased mortality. However, when JDP2 was overexpressed for 10 weeks, four out of 15 mice died, thereby revealing a mortality rate of 27%, whereas survival of time-matched WT mice did not decline. The cause of death was not analyzed in JDP2 mice, nor were ECG recordings performed. However, the observed development of impaired ventricular function within 5 weeks of JDP2 overexpression indicate that those mice probably died of sudden cardiac death or heart failure due to disease progression within 10 weeks.

### 2.2. Atrial Hypertrophy

**Figure 2:**
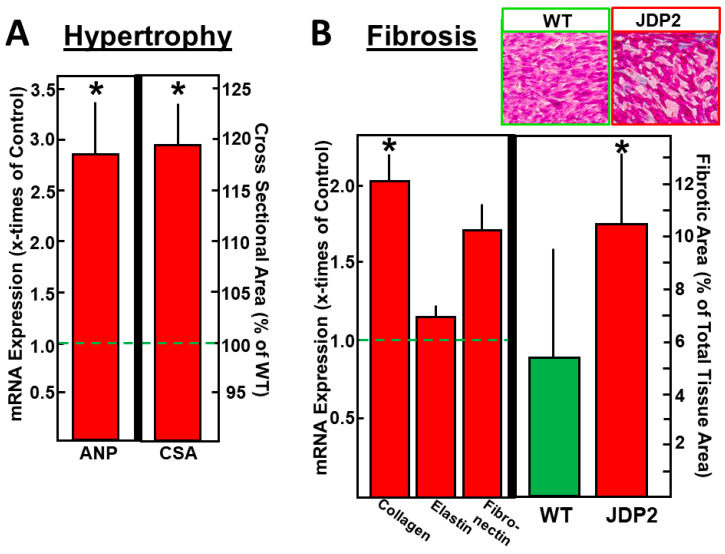
Hypertrophy and fibrosis were analyzed in atria after five weeks of JDP2 overexpression. (**A**) mRNA expression of the hypertrophic marker gene ANP was determined by real-time RT-PCR and compared to 18SrRNA as a housekeeping gene (*n* = 11–12, *p* < 0.05 vs. WT) and a cross-sectional area of isolated cardiomyocytes was calculated from 30–40 cells per preparation (values are means ± SD of 4 independent culture preparations). (**B**) mRNA expression of fibrotic marker genes was determined by real-time RT-PCR and compared to 18SrRNA as a housekeeping gene (*n* = 11–12, *p* < 0.05 vs. WT), and fibrotic areas were determined after staining atrial tissue sections with azan dye (values are means ± SD of 5–6 tissue sections). * Difference from WT, *p* < 0.05.

As already described recently, we and others have observed atrial dilatation after JDP2 overexpression for 4 or 5 weeks [9,10]. This increased atrial size may be caused by atrial cardiomyocyte hypertrophy and/or atrial fibrosis. In order to define the main reason for atrial dilatation, we first determined mRNA expression of hypertrophic and fibrotic marker genes. After 1 week of JDP2 overexpression, no changes in mRNA expression were observed. However, after 5 weeks the hypertrophic marker gene ANP (atrial natriuretic peptide) was 2.9 times higher than in WT mice (*n* = 11–12, *p* <0.05) (Figure 2A), and mRNA expression of the fibrotic marker gene collagen1 was increased by 2.0 times (*n* = 11–12, *p* < 0.05), whereas the expression of elastin and fibronectin mRNA remained unchanged (Figure 2B). The percentage of fibrotic areas in histological atrial sections increased (10.6% ± 2.5% in JDP2 mice vs. 5.2% ± 4.3% in WT mice, *n* = 5–6, *p* < 0.05). The cross-sectional area of atrial cardiomyocytes isolated from JDP2 mice was increased by 19.2% ± 4.0% (*n* = 4, *p* < 0.05, Figure 2A).

### 2.3. Inflammatory Parameters Are Increased in JDP2 Mice

**Figure 3:**
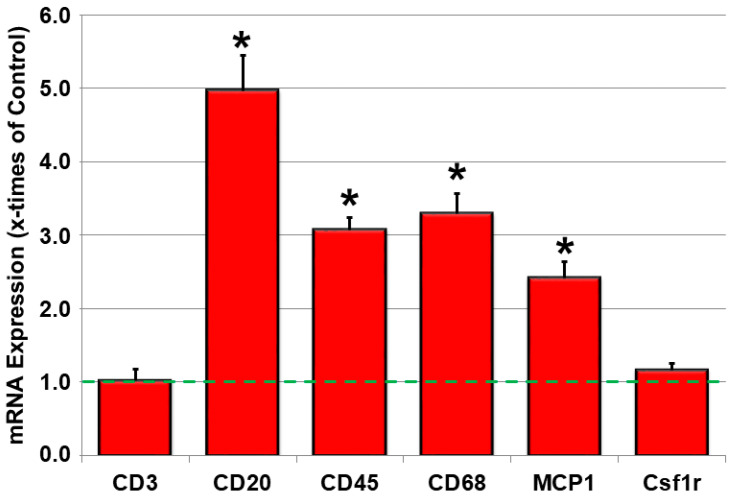
Atrial inflammation contributes to AF-associated electrical remodeling in JDP2-overexpressing mice. mRNA expression of the inflammatory marker genes in atria of mice overexpressing JDP2 for 5 weeks were determined by real-time RT-PCR and compared to 18SrRNA as a housekeeping gene (*n* = 7–12, * *p* < 0.05 vs. WT).

Since inflammatory cells may contribute to atrial structural remodeling and promotion of arrhythmia-inducing atrial substrates, we were interested in the spectrum of immune cells that may accumulate in the arrhythmic heart. Therefore, mRNA expression of markers for different immune cells were analyzed. As depicted in Figure 3, leukocyte common antigen (CD45), a common marker of immune cell infiltration, was enhanced 5 weeks after JDP2 overexpression. The main contributing inflammatory cells were monocytes/macrophages, since CD68 mRNA increased in JDP2 mice, as well as CD20/B-lymphocytes, since CD20 mRNA was increased 5.0 times compared to WT mice (*n =* 7–10, *p* < 0.05), whereas CD3/T_H_-lymphocytes mRNA and colony stimulating factor 1 receptor (Csf1r) mRNA remained unchanged. Furthermore, the inflammatory cytokine monocyte chemoattractant protein-1 (MCP-1) was increased by 2.4 times compared to WT mice (*n* = 7–10, *p* < 0.05).

### 2.4. Dysregulated mRNA Expression of Calcium-Handling Proteins and Connexin40 in JDP2 Mice

**Figure 4:**
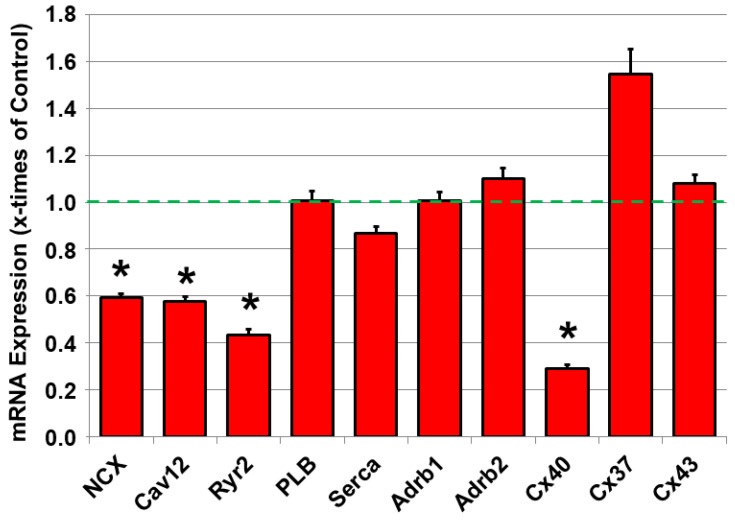
mRNA expression of connexins, β-adrenergic receptors and calcium-handling proteins. mRNA expression of connexins (Cx40, Cx37, Cx43), β-adrenergic receptors (Adrb1, Adrb2) and calcium-handling proteins (NCX, Cav1.2, Ryr2, PLB, Serca) in atria of mice overexpressing JDP2 for 5 weeks were determined by real-time RT-PCR and compared to 18SrRNA as a housekeeping gene (*n =* 11–12,* *p* < 0.05 vs. WT).

Since calcium handling and intercellular communication are decisive factors in the generation and propagation of action potentials and the contractility of atrial cardiomyocytes, the mRNA expression of calcium-handling proteins and beta-adrenoceptors was analyzed, as they augment calcium signaling, as well as connexins in the atrial tissue. As depicted in Figure 4, mRNA of beta-adrenoceptors AdRb1 and 2 did not differ from those in WT mice. However, reductions in the mRNA expression of NCX, Cav1.2 and RyR2 were observed after 5 weeks of JDP2 overexpression, as well as connexin40 mRNA (*n* = 11–12, *p* < 0.05 vs. WT), whereas connexins37 and 43 did not change compared to WT mice (Figure 4).

### 2.5. Remodeling of Sarcoplasmic Reticulum Calcium Handling in JDP2 Mice

**Figure 5:**
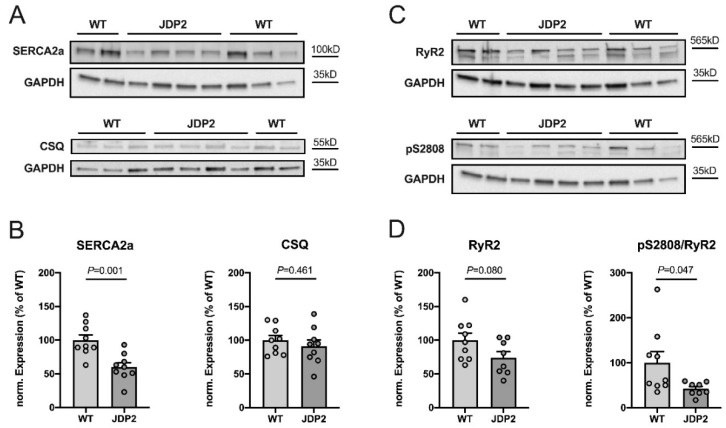
Expression and phosphorylation of sarcoplasmic reticulum (SR) calcium-handling proteins in atria from JDP2-overexpressing mice. Original Western blots (**A**,**C**) and summarized data (**B**,**D**) from 8–9 atria isolated from mice overexpressing JDP2 for 5 weeks vs. WT mice (*p*-values as indicated). Expression of SERCA2a, CSQ and RyR2 was analyzed, as well as phosphorylation of RyR2 at S2808. GAPDH was used as loading control for normalization. Note that the GAPDH signals shown in (**A**) for SERCA2a and in (**C**) for pS2808 are identical, as the proteins were derived from the same Western blot.

Using Western blotting, we analyzed whether the expression or phosphorylation of sarcoplasmic reticulum (SR) calcium-handling proteins were altered in JDP2 mice. Figure 5A,C illustrates the original Western blots, whereas B and D show the summarized results. Expression of SERCA2a, the SR-Ca-ATPase responsible for sarcoplasmic reticulum (SR) calcium reuptake in the diastole, was greatly reduced in JDP2 atria to ≈60% of the levels found in WT mice (*n =* 9, *p* = 0.001 vs. WT). By contrast, the expression of calsequestrin (CSQ), an SR calcium-buffering protein, was unaffected by JDP2 overexpression (*n =* 9, *p* = 0.461 vs. WT). RyR2 is the predominant cardiac SR calcium release channel. It is highly regulated by phosphorylation, with S2808 being one of the major phosphorylation sites. As shown in Figure 5C,D, expression of RyR2 tended to be reduced in JDP2 mice, but the difference in comparison to WT mice did not reach statistical significance (*n =* 8–9, *p* = 0.080 vs. WT). Phosphorylation of RyR2 at S2808, however, was significantly reduced in JDP2 mice (*n =* 8–9, *p* = 0.047). Thus, JDP2 mice exhibit remodeling of SR calcium handling, with reduced expression of SERCA2a and reduced phosphorylation of RyR2 at S2808, suggesting altered SR calcium release and reuptake.

## 3. Discussion

Atrial fibrillation (AF) is one of the most common arrhythmias in humans, and due to our aging population, increasing patient numbers can be expected in upcoming years. Furthermore, due to the multifactorial nature of atrial remodeling, it is difficult to pinpoint an initial trigger for AF. The most common therapy options are cardioversion or catheter ablation in combination with antiarrhythmic drugs [1]. Unfortunately, not all patients can be treated successfully. Therefore, a better understanding of the molecular and cellular mechanisms underlying AF development is urgently needed to define new therapeutic options or to improve existing techniques for the assessment and management of invasive and non-invasive procedures for these patients.

For this purpose, it is necessary to define animal models that resemble the development of spontaneous AF in humans. Many studies that tried to define such animal models in mice have been confronted with the problem that AF did not develop spontaneously [6]. Although structural alterations like atrial dilation or fibrosis were observed, AF had to be induced by an electrical burst, though it was not present without this treatment. Furthermore, derivation of ECGs under anesthesia may induce AF, because many anesthetics are known to facilitate the onset of AF [6]. For this reason, we have demonstrated here that JDP2 overexpression produces spontaneous paroxysmal AF under roaming conditions. For this analysis, we used a system that enabled ECG recordings to be performed non-invasively on conscious mice in order to detect the spontaneous appearance of AF in mice. ECG recordings were performed weekly, and after 4 and 5 weeks of JDP2 overexpression, periods of spontaneous paroxysmal AF were detected.

Mice with heart-specific overexpression of CREM-Ib∆CX resemble AF induction in our JDP2-overexpressing mice [13]. Both JDP2 and CREM-Ib∆CX are inhibitors of transcription factors. In part, the specificities of JDP2 and CREM-Ib∆CX overlap, as they both inhibit transcriptional processes that are promoted from CRE-promoter elements. CREB and AP1 are transcription factors that bind to CRE elements [14]. In contrast to CREM-Ib∆CX, JDP2 is not restricted to the inhibition of CRE-promoted transcription but can also interfere in nucleosome assembly or modulate the recruitment of multiple members of histone deacetylases [15]. Therefore, the spectrum of transcriptional regulation by JDP2 is broader in scope. Depending on the genetic background (FVB/N or FVB/N:CD-1) and on the heterozygous or homozygous expression of CREM-Ib∆CX, transgenic mice developed atrial enlargement and AF within 6 to 16 weeks [13,16]. A clear gene dose effect could be seen—higher amounts of the inhibitor resulted in faster AF development. JDP2 mice were both homo- and heterozygous. This may be the reason why we did not detect AF in all JDP2 mice within 5 weeks of JDP2 overexpression.

In spite of all these similarities between CREM-Ib∆CX and JDP2 mice, one major difference is the development of ventricular dysfunction in JDP2 mice, whereas in CREM-Ib∆CX no change or even improvements in ventricular function were observed. Ventricular dysfunction in JDP2 mice precedes atrial conduction defects or atrial expression changes. Whether the preceding ventricular impairments trigger or influence atrial dysfunction or whether these effects are independent of each other cannot yet be decided. However, since ventricular dysfunction is often a prerequisite of AF development in humans, JDP2 mice offer the potential to be an interesting model to study the mechanisms of AF induction in the context of ventricular impairments.

In JDP2 mice, AF developed in the presence of complicating structural abnormalities such as atrial dilatation and atrial hypertrophy, developing as a result of enlarged cardiomyocytes as well as increased collagen1 expression and fibrosis. Interpolating collagen strands may impair intercellular side-to-side myocyte connections, resulting in electrical decoupling that retards transverse wavefront propagation, thus enhancing functional re-entry [17]. Decoupling of cardiomyocytes may be augmented by downregulation of the gap junction-forming protein connexin40 in JDP2 mice. Calcium-handling remodeling occurs in many—if not all—models of AF investigated so far, including human AF. It may be either a contributing factor triggering episodes of AF or a consequence of AF-induced remodeling processes, or both [2,3]. In JDP2 mice, we have identified reduced expression of SERCA2a and reduced phosphorylation of RyR2 at S2808, as major calcium handling remodeling processes likely altering sarcoplasmic reticulum (SR) calcium release. The latter observation contrasts with chronic AF in humans, in which increased RyR2 phosphorylation is observed consistently [18]. Reduced expression of SERCA2a and at least unaltered phosphorylation of RyR2, however, were found in human paroxysmal AF and in rats with hypertensive heart disease and heart failure [19,20]. In both cases, these alterations (among others) were associated with increased proarrhythmic alterations in SR calcium release.

Another potential explanation for how JDP2 overexpression could induce changes in structural remodeling and thus predispose patients to AF provided by this murine model is atrial inflammation. It is not known whether the inflammatory cells are a marker of local reaction to tissue injury caused by factors leading to AF or whether they actively participate in the maintenance of AF due to direct cytotoxic or pro-fibrotic effects [5]. A plethora of blood-derived inflammatory biomarkers engaged in the pathogenesis of AF have been investigated, and many have proven to be associated with AF or its sequelae [5]. In addition to the analysis of calcium handling or interstitial fibrosis as triggers for AF, infiltration of the myocardium by monocytes/macrophages may serve as a potential trigger for AF [21]. Indeed, Yamashita et al. detected increased adhesion of CD45-positive cells in atrial specimens of AF patients, most of them being CD68-positive macrophages that apparently transmigrated into the myocardium [22]. This indicates a local immunologic inflammatory response with increasing numbers of resident immune cells. Furthermore, monocyte chemoattractant protein-1 (MCP-1), a proinflammatory chemotactic cytokine, which is synthesized in monocytes and macrophages along with other chemokines, is upregulated in patients with AF [21]. MCP-1 is involved in regulating the production of several matrix metalloproteinases. In JDP2 mice, we observed an enhancement of CD68 expression, a marker of monocyte/macrophage infiltration, and MCP-1, a cytokine which is released from circulating monocytes. Whether local inflammation of immune cells is a cause or a result of AF has not yet been clarified and should be a subject of future studies.

In conclusion, JDP2 overexpression causes atrial structural, pro-inflammatory and calcium handling remodeling, thus promoting the initiation of paroxysmal AF in mice. JDP2-overexpressing mice, therefore, represent a uniquely suited animal model for the investigation of mechanisms underlying the development of paroxysmal AF. Moreover, our results suggest that JDP2- and JDP2-regulated genes may be a novel therapeutic target for AF.

## 4. Limitations

Although we have identified several changes in atria of JDP2 mice, such as inflammation, hypertrophy, fibrosis and altered calcium handling, further studies are needed to clarify their mechanistic role(s) in the development of AF in JDP2 mice.

## 5. Methods

### 5.1. JDP2-Overexpressing Mice

Our investigation conformed to Directive 2010/63/EU of the European Parliament. Animal studies were approved by the Regierungspräsidium Gießen (registration-no. V54-19c20-15(1) GI20/1 Nr. 54/2008). JDP2 mice, a result of the crossbreeding of C57BL/6 J and FVB/N mice, were generated in Haifa, Israel. They are double-transgenic, carrying a heterozygote JDP2 gene (tetO) with a minimal promoter and a heterozygous transactivator gene (tTA) under the control of the cardiac-specific α-MHC promoter.

The transactivator can be regulated by the antibiotic doxycycline (Dox) in a Tet-Off system. Feeding animals with Dox blocks the interaction between the transactivator and the promoter of the JDP2 gene, thereby preventing JDP2 overexpression. To suppress JDP2 overexpression during the embryonic and juvenile development of mice, breeding pairs and newborn mice were fed with Dox for the first 4 weeks of their life. Then, mice were fed with an Altromin standard diet for up to 5 weeks, resulting in JDP2 overexpression. Genotypes of JDP2 mice were tTA/tetO. JDP2 overexpression was confirmed by real-time RT-PCR. Only transgenic mice (TM) with upregulated JDP2-mRNA (compared to WT) were considered to be JDP2-overexpressing mice and were incorporated in this study. Littermates of TM which did not overexpress JDP2 (wild types, WT) were used as controls. Thus, the WT group included the following genotypes—0/0, tTa/0 and tetO/0. WT mice received the Dox-diet for the same duration as the JDP2 mice.

### 5.2. Electrocardiography Recordings

ECGs were recorded non-invasively on conscious mice using the ecgTUNNEL-system (emkaTechnologies S.A.S, Falls Church, VA, USA), as described by others [23,24]. After the start of JDP2 overexpression, ECGs were recorded once every week for up to 5 weeks. The recording time was 30 min per session. Post-acquisition ECG analysis, based on a shape recognition technique, was carried out using ecgAUTO software (emkaTechnologies S.A.S, Falls Church, VA, USA). Heart rate variability was calculated from the RR values as the ratio of standard deviation/mean. Atrial fibrillation (AF) was defined by the absence of P-waves in combination with increased heart rate variability.

### 5.3. Atrial Tissue Preparation

Mice were euthanized by means of isoflurane inhalation (5% isoflurane) and cervical dislocation. Hearts were directly excised and blood was washed out with ice-cold 0.9% NaCl. Afterwards, atria were dissected from whole heart preparations in an ice-cold saline solution. Tissue was either shock-frozen in liquid nitrogen and stored at −80 °C or frozen tissue sections were made. Atrial tissue was used for real-time RT-PCR and Western blots.

### 5.4. Isolation of Atrial Cardiomyocytes and Determination of Cell Size

Mice were anaesthetized by means of isoflurane inhalation. After cervical dislocation, hearts were extracted, and retrogradely perfused in a Langendorff apparatus with a collagenase-containing calcium-free buffer equilibrated at 37 °C, pH 7.4. Atria were dissected from ventricles and atrial cardiomyocytes were separated from other cells by centrifugation. The medium was readjusted to a physiological calcium concentration and suspended in a basal culture medium. Cardiomyocytes were then plated on laminin-coated culture dishes. After 2 h, the medium was changed. The basal culture medium (CTT) was a modified medium 199 including Earl’s salts, 2 mM L-carnitine, 5 mol/L taurine, 100,000 IU/L penicillin, 100 mg streptomycin and 10 μmol/L cytosine-beta-D-arabinofuranoside. Myocyte size was determined on micrographs digitalized by a charge-coupled device camera. A total number of 30–40 rod-shaped cells of one preparation were measured. Width/diameter of myocytes was determined at the widest point of each myocyte using the software program Analysis from SIS. Cross-sectional area of cardiomyocytes was calculated using the following formula: (radius)^2^ × *p*.

### 5.5. Real-Time RT-PCR

Total RNA from atria was extracted using Trizol (Invitrogen) as described by the manufacturer. This was followed by DNAse treatment and reverse transcription with the QuantiTect Reverse Transcription Kit from Qiagen. For each assayed gene, the annealing temperature and the number of cycles resulting in a linear amplification range were tested. Real-time RT-PCR was performed in an automated thermal cycler and was detected with the Biorad detection system (Biorad) using SYBR Green fluorescence for quantification. The calculations of the results were carried out according to the 2^−ΔΔCt^ methods, as previously described [25]. Gene expression was compared to 18S ribosomal RNA (18SrRNA) as a housekeeping gene. Primer sequences are listed in Table 2.

### 5.6. Western Blots

Immunoblotting of atrial tissue lysates (14 μg protein per sample) was performed as described previously [20]. Proteins were separated on 4%–20% gradient gels (4%–20% Mini-PROTEAN^®^ TGX^TM^ Precast Protein Gels, #4561093, BIO-RAD, Feldkirchen, Germany). In order to detect various, differently sized proteins from a single membrane (and hence save sample material), membranes were cut between protein bands of interest. The resulting parts of the membrane were then probed with the antibody of interest. The following primary antibodies were used: anti-Ryanodine Receptor (C3-33) (#MA3-916, Thermo Fisher, Schwerte, Germany, 1:5000), anti-Ryanodine Receptor 2 (pSer2808) pAb (#A010-30, Badrilla, Leeds, UK, 1:5000), anti-SERCA2a pAB serum (#A010-20, Badrilla, 1:5000), anti-Calsequestrin Polyclonal Antibody (#PA1-913, Thermo-Fisher, 1:2500) and anti-GAPDH (mAb (6C5), Calbiochem, Darmstadt, Germany, 1:50.000). GAPDH was used for normalization of expression.

### 5.7. Analysis of Fibrosis

Whole hearts were embedded in “Tissue Freezing” medium (R. Langenbrinck) in cryomold bowls (Weckert Labortechnik). Then, samples were snap-frozen in 2-methylbutane-isopentan (Fluka). Frozen tissues were cut into slices and stained with hematoxylin-eosin to detect muscle tissue in red and azan dye to detect collagen in blue. Micrographs were taken, and fibrotic areas were determined using ImageJ software.

### 5.8. Statistical Analysis

If not indicated otherwise, data are presented as mean ± SEM. Normally distributed data were analyzed with an unpaired 2-tailed Student *t*-test or ANOVA, nonnormally distributed data with the Mann–Whitney U test or Kruskal–Wallis ANOVA. Analyses were performed using SigmaPlot (Systat Software, Erkrath, Germany), GraphPad Prism 8 and SPSS. A *p*-value <0.05 was considered statistically significant.

## Figures and Tables

**Table 1:** ECG recorded time intervals in WT and JDP2 mice. ECGs were recorded non-invasively on conscious mice 1 and 5 weeks after JDP2 overexpression for 30 min. Post-acquisition ECG analysis was based on a shape recognition technique. Heart rate variability was calculated from the RR-values as a ratio of standard deviation/mean. * *p* < 0.05 vs. WT from *n =* 6–11 animals.

ECG Recordings	1 Week		5 Weeks	
	WT	JDP2	WT	JDP2
P-wave duration	10.0 ± 0.9 ms	10.6 ± 0.8 ms	10.0 ± 1.5 ms	11.6 ± 1.1 ms *
PQ-interval	32.0 ± 5.1 ms	32.3 ± 2.6 ms	30.3 ± 7.0 ms	35.1 ± 3.6 ms
QRS-complex	14.3 ± 1.2 ms	13.0 ± 1.2 ms	13.9 ± 1.7 ms	15.8 ± 2.3 ms *
RR-interval	86.8 ± 5.0 ms	84.9 ± 4.0 ms	83.3 ± 4.1 ms	84.5 ± 3.1 ms
HR-variability	0.031 ± 0.013	0.042 ± 0.034	0.021 ± 0.01	0.027 ± 0.01
QT-interval	29.1 ± 0.7 ms	29.1 ± 1.4 ms	29.5 ± 1.8 ms	31.3 ± 1.9 ms
*n*	6	7	10	11

**Table 2:** Primer sequences for real-time RT-PCR.

Primer	Sequences of Primer
Adrb1	Qiagen, QT00258692
Adrb2	5′-TGGTACCGTGCCACCCACAA-3′
	5′-AAGACCATCACCACCAGGGGCA-3′
ANP	5′-CTGCTAATCAGCCATGCAAA-3′
	5′-GATGGAGACCATCCTGGCTA-3′
Cav1.2	5′-CAGCCACTCTCCAGTCACTC-3′
	5′-CTGGAGTAGGGATGTGCTCG-3′
CD3	5′-ATGCGGTGGAACACTTTCTGG-3′
	5′-GCACGTCAACTCTACACTGGT-3′
CD11b	5′-CTGAGACTGGAGGCAACCAT-3′
	5′-GATATCTCCTTCGCGCAGAC-3′
CD20	5′-CCTTTCCCAGCAGAGCCTAC-3′
	5′-TCATGATTTGGACAGCCCCC-3′
CD45	5′-ATGGTCCTCTGAATAAAGCCCA-3′
	5′-TCAGCACTATTGGTAGGCTCC-3′
CD68	5′-ACTTCGGGCCATGTTTCTCT-3′
	5′-GCTGGTAGGTTGATTGTCGT-3′
Collagen1	5′-TTCTCCTGGRAAAGATGGTGC-3′
	5′-GGACCAGCATCACCTTTAACA-3′
Csf1r	5′-TCCACCGGGACGTAGCA-3′
	5′-CCAGTCCAAAGTCCCCAATCT-3′
Cx37	5′-ATAAAGGCACGAAGGGACCA-3′
	5′-GTCAAGTTGGCCCAGTTCTG-3′
Cx40	5′-AGGGCTGAGCTTGCTTCTTA-3′
	5′-TTAGTGCCAGTGTCGGGAAT-3′
Cx43	5′-GAAACAATTCCTCCTGCCGC-3′
	5′-AGTTGGAGATGGTGCTTCCG-3′
Elastin	5′-CTGCTGCTAAGGCTGCTAAG-3′
	5′-CCACCAACACCAGGAATGC-3′
Fibronectin	5′-ACAGAGCTCAACCTCCCTGA-3′
	5′-TGTGCTCTCCTGGTTCTCCT-3′
JDP2	5′-ATGATGCCTGGGCAGATCCCA-3′
	5′-TCACTTCTTGTCCAGCTGCTCC-3′
MCP1	5′-CCACAACCACCTCAAGCA-3′
	5′-TGAAAGGGAATACCATAACATC-3′
NCX	5′-CTACCAGGTCCTAAGTCAACAG-3′
	5′-TGCGTGCCTCTTCAAGATG-3′
PLB	5′-GCAATACCTCACTCGCTCGGCTATC-3′
	5′-TGGAGATTCTGACGTGCTTGCTGAG-3′
RyR	5′-AGCTGGAAGACCCTGCAATC-3′
	5′-ACCAGGCTGAAATATCCCCG-3′
SERCA	5′-TGACTGGTGATGGTGTGAATG-3′
	5′-GATGAGGTAGCGGATGAACTG-3′
18SrRNA	5′-TTGACGGAAGGGCACCACCA-3′
	5′-AGAACGGCCATGCACCACCA-3′
